# Clinical Cancer and Direct-to-Consumer Genetic Test Result-Sharing Behavior: Findings from HINTS 2020

**DOI:** 10.3390/jpm13010018

**Published:** 2022-12-22

**Authors:** Sukh Makhnoon, Robert Yu, Susan K Peterson, Sanjay Shete

**Affiliations:** 1 Department of Behavioral Science, The University of Texas MD Anderson Cancer Center, Houston, TX 77030, USA; 2 Department of Biostatistics, The University of Texas MD Anderson Cancer Center, Houston, TX 77030, USA; 3 Department of Epidemiology, The University of Texas MD Anderson Cancer Center, Houston, TX 77030, USA; 4 Division of Cancer Prevention and Population Sciences, The University of Texas MD Anderson Cancer Center, Houston, TX 77030, USA

**Keywords:** communication, result sharing, genetic testing, cancer, direct-to-consumer

## Abstract

Introduction: Sharing genetic test results with different stakeholders such as family members, healthcare providers and genetic counselors (HCP/GCs), spouses/partners, and friends is a health behavior of clinical importance in genomic medicine. Methods: Using nationally representative population-based data collected from the Health Information National Trends Survey (HINTS 5, cycle 4), we identified the prevalence and factors associated with genetic test result-sharing behavior for high-risk cancer tests, genetic health risk tests, and ancestry tests within four groups: HCP/GCs, first-degree relatives (FDRs), spouse/partner, and friend/other. Results: Overall, 68.4% of those who underwent high-risk cancer genetic testing shared their results with FDRs, whereas 89.9% shared with HCP/GCs. In adjusted multivariable analyses, women were nine times more likely than men to share (*p* = 0.006), and those with a personal history of cancer were less likely to share with HCP/GCs (OR = 0.025, *p* ≤ 0.001). Of those tested for genetic health risk, 66.5% shared with HCP/GCs, 38.7% with FDRs, 66.6% with a spouse/partner, 12.8% with a friend, and 14.1% did not share results with anyone. Of those who underwent ancestry testing, very few shared results with HCP/GCs (2.6%), whereas modest sharing was reported with FDRs, spouses/partners, and friends. Discussion: These data add empirical evidence about the population prevalence of genetic information sharing and serve as a metric for public engagement with genetic testing.

## 1. Introduction

Sharing genetic test results is a health behavior of much interest in genomic medicine. Genetic tests currently available to most individuals include multigene panels that can identify inherited susceptibility to cancer, cardiovascular disease, and a range of other disorders [1,2]. Outside the healthcare setting, common tests performed through direct-to-consumer (DTC) genetic testing (e.g., 23andMe, Ancestry) include ancestry testing and limited health risk tests. Less commonly available tests include exome and genome sequencing, where a vast number of genes are queried for relevance to the phenotype of interest [3]. The sharing of each of these types of genetic test results with healthcare providers, family members, a spouse, or friends has unique implications in genomic medicine. 

Sharing behavior is perhaps most salient for results of high-risk cancer testing, which should be shared with family members because the information can inform relatives’ decisions regarding health risks, cascade genetic testing [4], and subsequent genetically informed disease prevention through increased surveillance or surgery [5]. It is also important, and often necessary, to share genetic health risk results with healthcare providers, as tests are often performed in siloed specialized centers that do not share results with patients’ other healthcare providers, e.g., primary care physicians. Sharing results with a spouse/partner can be important as they often play a key role in facilitating family communication of genetic information, which is necessary for eventual cascade testing [6]. Sharing with friends and other individuals has few direct health implications but may still be important for raising awareness about genetic testing [7].

Sharing results from consumer-driven, physician-mediated DTC genetic testing is also worthy of serious attention. It is estimated that as of 2019, more than 26 million people worldwide have undergone DTC genetic testing [8]. Although some results of these tests can have health implications (e.g., testing for three *BRCA1/2* variants), most tend to be peripherally or not related to healthcare, such as the propensity for dry or wet earwax. Still, the public appetite for these tests is worth investigating as these tests have been suggested as a way to address access-to-care gaps [9] and may affect patients’ attitudes toward clinical genetic testing. Actionable genetic health risk results from DTC settings must be shared with healthcare providers in order to confirm the results in a CLIA-certified laboratory and to inform subsequent healthcare management. The sharing of DTC ancestry results with family members or healthcare providers is likely less important in medicine today but may grow in relevance as the field learns to apply ancestry data in routine medical care. Nevertheless, the behavior of sharing ancestry test results can serve as a metric of lay people’s engagement with and interest in genomics, which is key to the subsequent utilization of clinical genetic services. 

The behaviors of sharing results with different types of individuals have been studied largely as secondary outcomes of genetic research studies [10,11,12]. Health communication research in this area suggests that sharing with relatives is often incomplete and riddled with barriers [11]. Several reasons have been given for not informing adult relatives about genetic testing results, including estrangement, family disruption, feeling that the recipient did not have sufficient maturity [13,14], and a desire to spare others the sorrow brought by information that might be painful. Sometimes tested individuals have been found to be unaware of their relatives’ elevated health risk associated with their own test results, so it is not surprising that behaviors of sharing test results are affected by difficulties with understanding complex genetic and medical information [15]. 

Population-level data on result sharing is sparse and limited by their lack of differentiation among types of genetic tests. A 2015 study used population-level data to understand *BRCA1/2* and Lynch syndrome genetic test result-sharing behavior and found high result-sharing with healthcare professionals and family members [16]. However, sharing of DTC results was not reported. Given the increased awareness and use of genetic testing over time, it is necessary to update these data and expand on the findings. 

To address this gap in research, we used nationally representative population-based data collected from the Health Information National Trends Survey (HINTS 5, cycle 4) to report the prevalence of and factors associated with genetic test result-sharing behavior for high-risk cancer tests, genetic health risk tests, and ancestry tests to four different groups of individuals: healthcare providers, first-degree relatives (FDRs), spouse/partner, and friend/other. Due to the small sample sizes in some categories, these data should be considered exploratory.

## 2. Materials and Methods

Study participants were respondents of HINTS: a nationally representative cross-sectional survey administered every few years in the United States by the National Cancer Institute. The fifth iteration of this survey (HINTS 5) started in 2017, and data from the latest data collection cycle (cycle 4) were used in this analysis. HINTS5, cycle 4, was fielded between February and June 2020 with a response rate of 36.7%. The survey’s content focused on genetic testing and health-related knowledge, perceptions, and behaviors, as well as the accessibility, need for, and use of health-related information. A detailed overview of the study design is available elsewhere [17,18]. Detailed methodological information is provided online [19]. Briefly, this self-administered mailed questionnaire was sent to U.S. addresses in two strata: those in areas with high concentrations of minority populations and those in areas with low concentrations of minority populations. Surveys were available in English and Spanish; a $2 monetary incentive was included with the survey to encourage participation. Our study used data from a publicly available, anonymized database and therefore was exempt from ethical compliance and oversight by an institutional review board.

### 2.1. Measures

#### 2.1.1. Genetic Test Result Sharing

Genetic test information was obtained from the question, “Have you ever had any of the following types of genetic tests?” with the answer choices of ancestry testing, genetic health risk testing, high-risk cancer testing, other, not sure, and none of the above. Genetic health risk testing was defined as a test “to determine health risk for a variety of health conditions (for example, 23andMe),” high-risk cancer testing was defined as a test for “(for example, *BRCA1/2* or Lynch syndrome),” and ancestry testing was defined as a test “to determine the background or geographical/ethnic origin of an individual’s ancestors (for example, Ancestry.com and 23andMe).” The present analysis focused on high-risk cancer testing because of its strong relevance to healthcare. 

Data on sharing test results were obtained from the question, “If you had a genetic test, who did you share the results with?” with options to choose from one or more of the following responses: your health care provider, genetic counselor, spouse/partner, parents, siblings, children, friend, other, and did not share the results. Healthcare providers and genetic counselors were categorized as HCP/GCs; parents, siblings, and children were categorized as FDRs. 

Based on prior research on predictors of genetic test result sharing, we included a number of sociodemographic, clinical, and psychosocial variables in our exploratory analysis.

#### 2.1.2. Clinical Factors

Family (defined as first- and second-degree biological relatives) and personal history of cancer were assessed using the following items: “Have any of your first- or second-degree biological relatives (parents, brothers and sisters, children, grandparents, aunts and uncles, nieces and nephews) ever had cancer?” (yes, no, not sure) and “Have you ever been diagnosed as having cancer?” (yes, no) with a follow-up question that provided 23 specific cancer types to choose from. 

#### 2.1.3. Risk Perceptions

Perceived genetic susceptibility to cancer was measured using one item that asked, “How much do you think genes that are inherited determine whether or not a person will develop [cancer]?” The role of genetics in cancer prevention and early detection was measured using two items, “How important is knowing a person’s genetic information for: a) preventing cancer; and, b) detecting cancer early?” For all risk perception questions, response options ranged from 1 (a lot) to 4 (not at all). Responses were dichotomized as not at all/a little vs. very/somewhat for analysis.

#### 2.1.4. Cancer Beliefs

Cancer worry was measured using the item, “How worried are you about getting cancer?” Response options ranged from 1 (not at all) to 5 (extremely) and were trichotomized as high cancer worry (extremely/moderately), moderate cancer worry (somewhat), and low cancer worry (not at all/slightly). Additional potentially relevant beliefs were assessed using participants’ agreement with two items, “There’s not much you can do to lower your chances of getting cancer” and “There are so many different recommendations about preventing cancer, it’s hard to know which ones to follow,” on a scale of 1 (strongly agree) to 4 (strongly disagree). Cancer fatalism was assessed using the item, “It seems like everything causes cancer,” with response options ranging from 1 (strongly agree) to 4 (strongly disagree). Responses to the last three items were dichotomized as strongly agree/somewhat agree vs. somewhat disagree/strongly disagree for analysis. 

#### 2.1.5. Genetic Self-Efficacy

Self-efficacy regarding the ability to engage in behavior change based on genetic information related to cancer was assessed using the item “If I found out from a genetic test that I was at high risk of cancer, I would change my behaviors such as diet, exercise and getting routine medical tests,” with response options ranging from 1 (strongly agree) to 4 (strongly disagree). Desire to know about mutation status was measured using one item, “How much would you want to know if you have a genetic change that increases your chances of getting cancer?” with response options ranging from 1 (a lot) to 4 (not at all). Both responses were dichotomized for analysis. 

### 2.2. Statistical Analysis

We incorporated survey sampling weights specified for HINTS 5, cycle 4, into our analyses to account for the complex sampling framework used in the HINTS survey and to provide estimates representative of the U.S. population. Units of survey weights were inversely proportional to the probability of selection and response. The computation of full-sample weights included calculating the houshold-level base weights for each household in the sample, adjustments for non-response, initial person-level weight for the adult in each responding household, and calibration of survey weights to the American Community Survey 2018. Additional details about the specific weighting methodology can be found in the Methodology Report [19]. 

Survey-weighted prevalence of cancer genetic testing was calculated for the overall study sample and for groups after stratification by risk perceptions, cancer-related beliefs, and sociodemographic factors. We used the survey-weighted chi-squared test and t-test for bivariate analyses for sharing behavior by categorical variables and sharing behavior by age, respectively. We used survey-weighted multivariable logistic regression using the PROC SURVEYLOGISTIC procedure in the SAS/STAT software incorporating complex survey sample design accounting for strata, cluster, weights, and replicate weights with the jackknife variance estimation method to identify factors associated with genetic test result sharing. Pre-selected covariates, based on factors associated with genetic test result sharing in the literature [16,20,21], were included in the multivariable model. Statistical significance was determined using a two-sided *p*-value ≤ 0.05. Statistical analyses were conducted using survey analysis procedures SAS/STAT (version 9.4).

## 3. Results

Of the 3859 respondents to HINTS, 142 (3.3%) reported undergoing high-risk cancer genetic testing. Seventy-seven of these respondents were excluded as they did not respond to the question on sharing test results, leaving 65 respondents in the analysis of high-risk cancer genetic test result-sharing behavior. Most test takers were female (88.3%), non-Hispanic White (91.7%), insured (100%), had an annual household income of $75,000 or higher (41.3%), and resided in an urban zip code (81.8%). Of those who underwent high-risk cancer genetic testing, 94% had a first-degree family history of cancer, and 80% had a personal history of cancer. It is possible that individuals with a personal and family history of cancer met the referral criteria and, thus, were more likely to undergo testing. 

### 3.1. High-Risk Cancer Genetic Test Results 

**Figure 1:**
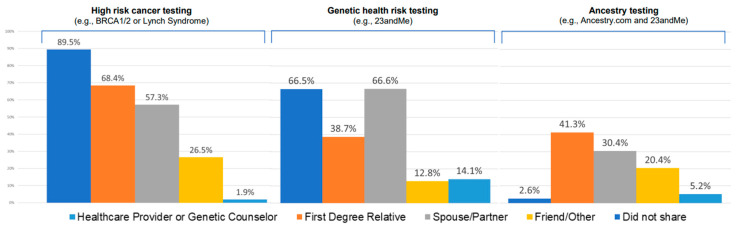
Sharing behavior of clinical and direct-to-consumer genetic test results. For each type of genetic test result, respondents answered the question, “…who did you share the results with?” Selecting multiple answers was allowed.

Overall, 89.9% of those who underwent high-risk cancer genetic testing shared their results with an HCP/GC (Figure 1). Sharers were more likely to be younger (50.1 vs. 59.1 years, *p* = 0.018), have ≤ high school education (80.1% vs. 46.6%, *p* = 0.036), have a personal history of cancer (92.8% vs. 29.5%, *p* ≤ 0.001), and reside in a rural area (80.1% vs. 96.9%, *p* = 0.023). Overall, most test takers rated the importance of genetics for cancer prevention as little or not at all, yet these respondents who believed that were more likely to share results with an HCP/GC (*p* = 0.019). Other sociodemographic and clinical characteristics were comparable between sharers and non-sharers (Table 1).

In the multivariable analyses adjusted for sex, race/ethnicity, income, family, and personal history of cancer, only sex and personal history of cancer were associated with sharing results with an HCP/GC. Female respondents were nine times more likely to share than male respondents (*p* = 0.006), and those with a personal history of cancer were less likely to share than those without (OR = 0.025, *p* ≤ 0.001) (Table 2).

Compared with the percentage who shared results with an HCP/GC, a smaller proportion (68.4%) of high-risk cancer testers shared results with an FDR. Those with a family history of cancer were more likely to share their results with FDRs (98.4% vs. 1.6%, *p* ≤ 0.001) than those without, but all other demographic, clinical, and psychosocial characteristics were similar between sharers and non-sharers (Table 1). In the adjusted multivariable analysis, we found no significant association between sociodemographic characteristics and sharing with an FDR (Table 2). Of all high-risk cancer genetic testers, 57.3% shared results with their spouse/partner, 26.5% with a friend, and 1.9% did not share results with anyone (Figure 1).

### 3.2. Genetic Health Risk Testing

Of all HINTS respondents, 5.3% (n = 233) underwent genetic health risk testing, and 1.9% (n = 71) responded to the question on sharing. Of the test takers, 66.5% shared with an HCP/GC, 38.7% with an FDR, 66.6% with a spouse/partner, 12.8% with a friend, and 14.1% did not share results with anyone (Figure 1). The distribution of sharing genetic health risk test results with HCP/GCs and FDRs by relevant sociodemographic characteristics is shown in Table 3.

### 3.3. Ancestry Testing

Of all respondents, 12.4% (n = 538) underwent genetic ancestry testing, and 7.1% (n = 303) responded to the question on sharing. Very few of these people shared their results with an HCP/GC (2.6%), whereas modest sharing was reported with an FDR, spouse/partner, or friend (41.3%, 30.4%, and 20.4%, respectively). Additionally, 5.2% of ancestry testers did not share results with anyone (Figure 1). The distribution of sharing ancestry test results with HCP/GCs and FDRs by relevant sociodemographic characteristics is shown in Table 4.

## 4. Discussion

Sharing genetic test results with various stakeholders is a health behavior of clinical relevance, as well as one that is key to improving public awareness of genetic testing. In this analysis of population-representative data on sharing clinical genetic test results, 90% of those who underwent high-risk cancer genetic testing (e.g., *BRCA1/2* and Lynch syndrome) shared results with HCP/GCs, whereas only 68% shared results with their FDRs. Our findings may help contextualize the feasibility of proband-mediated cascade genetic testing that traditionally relies on sharing genetic test results with relatives. The findings also provide empirical data on public engagement with different types of genetic tests and offer indications of how these results are used by test takers. 

Our finding that 90% of those who underwent high-risk cancer genetic testing shared results with HCP/GCs is an increase from the 73% sharing reported in 2015 [16]. Female participants in our study were more likely to share results with HCP/GCs, which may be explained by the higher use of cancer genetic testing for gendered conditions such as breast and ovarian cancers as well as the gender difference in healthcare utilization whereby women are more likely to consult healthcare providers [22]. However, given the large difference in sample size between sexes and large confidence intervals in our study, the results should be validated in future studies with larger sample sizes. Similar to prior research [23], we found that individuals without a personal history of cancer were more likely to share high-risk cancer results with an HCP/GC. This finding may be explained, in part, by the definition of HCP used by HINTS. This survey did not distinguish between sharing with primary care physicians, oncologists, or other specialty physicians, although there is evidence to indicate differential sharing by provider type among cancer patients [24]. Similar differences may exist among individuals without a personal history of cancer as recommendations from primary care physicians are known to influence patient adherence to cancer surveillance [25,26], and sharing genetic test results may be key to successful cancer risk management. A surprising finding from our bivariate analysis is that respondents who believed in the importance of genetic testing for cancer prevention were less likely to share results with HCP/GCs. In our prior work using the same HINTS data, beliefs about genetically informed cancer prevention versus early detection were not associated with genetic testing in predictable ways [27]. Another explanation is that respondents may have interpreted sharing as the initiation of sharing, the opportunities for which are rare in clinical oncology. Most genetic test results are disclosed by providers to patients, and as such, providers initiate the discussion, not patients.

In our limited sample of people who underwent high-risk cancer genetic testing, 68.4% shared their results with an FDR. This is lower than the 75% sharing reported in the 2015 HINTS survey [16] but well within the large variation in familial sharing data reported in the literature. Such variation may be explained by a number of individual- and family-level factors known to influence family communication, including actionability of the genetic test result, genetic knowledge, and closeness to relatives [11,12]. The decrease in familial sharing is concerning for cascade genetic testing and may indicate the need to reduce our reliance on family communication to achieve this outcome, which is of clinical and public health importance. 

Outside of high-risk cancer genetic testing, we found that 66% of those who underwent DTC genetic health risk testing shared the results with an HCP/GC, whereas only 2.6% of those who underwent ancestry testing shared the results with an HCP/GC. However, the rates of sharing these results with FDRs were similar—39% among genetic health risk testing and 41% for ancestry testing. To our knowledge, this is the first report of genetic test result-sharing behavior using population-representative data that extends beyond cancer genetic testing. A continuing concern in the field of genomic medicine is the integration of DTC genetic testing with medical practice and its effects on the healthcare system and healthcare utilization. It is estimated that nearly half of DTC consumers seeking clinical confirmation of their results might be wasting healthcare resources and healthcare providers’ time [28]. While the 66% of consumers of DTC health-risk testing who shared their results with HCP/GCs may appear to be considerable, they constitute a small proportion of the overall U.S. population. Moreover, the low percentage of DTC ancestry testing consumers who shared their results with an HCP/GC suggests a limited impact on the healthcare system. In fact, consumer participation in DTC genetic testing may positively affect public attitudes toward genetic testing and may help narrow the gaps in access to genomic medicine. 

While our study offers important empirical data on the population prevalence of genetic information sharing, a limitation of this study is the small sample sizes associated with various types of genetic testing, which prevented us from including additional variables in the logistic regression models. However, ours is the first study to provide sharing data related to two types of DTC genetic testing as well as sharing with spouses and friends, which are key for public engagement with genetics. Although respondents included in this analysis were largely female and non-Hispanic white, they are representative of consumers of genetic testing today, where structural and institutional barriers prevent other groups from accessing tests. With our use of survey weights, the results on sharing are still generalizable to the U.S. population. Due to the cross-sectional study design and lack of survey items on when genetic testing was performed, we cannot evaluate the timeframe over which sharing occurred. Although most high-risk cancer results are shared with relatives within weeks to months of testing, these patterns could be different for genetic health risk or ancestry testing. Since HINTS did not provide “not applicable” as an option on the survey, we do not know if respondents who did not report sharing with a stakeholder, e.g., a parent, did not so because they did not share genetic test results with a parent or they did not have a living parent to share it with. Future surveys on this topic should provide nuanced answer choices and probe answers with follow-up questions to better understand how genetic information is shared.

In conclusion, these population-level data regarding sharing different types of genetic test results add important empirical evidence on the population prevalence of genetic information sharing and serves as a metric for public engagement with genetic testing in the United States.

## Figures and Tables

**Table 1:** Descriptive characteristics of individuals who shared results from high-risk cancer genetic testing.

Variables	Category	Total	Sharing with HCP/GC			Sharing with FDR		
		N (wtd%)	Shared n (wtd%)	Did Not Share n (wtd%)	*p*-Value	Shared n (wtd%)	Did Not Share n (wtd%)	*p*
Sociodemographic factors								
Age, y	Mean, SD	NA	50.1, 2.2	59.1, 2.3	**0.018**	49.8, 2.9	53.7, 3.7	0.422
Sex	Male	7 (11.7)	4 (9.9)	3 (27.69)	0.075	3 (7.15)	4 (21.89)	0.161
	Female	56 (88.3)	47 (90.1)	9 (72.31)		39 (92.85)	17 (78.11)	
Income	Less than $20,000	7 (11.1)	5 (11.2)	2 (9.71)	NA	5 (7.51)	2 (19.64)	0.876
	$20,000 to less than $35,000	5 (3.9)	4 (3.7)	1 (5.72)		2 (3.60)	3 (4.52)	
	$35,000 to less than $50,000	4 (10.0)	4 (10.8)	NA		3 (9.35)	1 (11.48)	
	$50,000 to less than $75,000	13 (33.7)	9 (32.1)	4 (54.34)		8 (34.63)	5 (31.64)	
	$75,000 or more	31 (41.3)	28 (42.2)	3 (30.22)		23 (44.92)	8 (32.71)	
Education	≤High school	34 (77.0)	30 (80.1)	4 (46.62)	**0.036**	22 (77.17)	12 (76.77)	0.978
	>High school	27 (23.0)	20 (19.9)	7 (53.38)		19 (22.83)	8 (23.23)	
Race/Ethnicity	Non-Hispanic White	48 (91.7)	39 (91.3)	9 (95.29)	NA	34 (93.68)	14 (86.53)	NA
	Non-Hispanic Black	3 (2.6)	2 (2.3)	1 (4.71)		1 (0.61)	2 (7.55)	
	Hispanic/Latino	5 (4.8)	5 (5.3)	NA		3 (4.33)	2 (5.92)	
	Non-Hispanic other	1 (1.0)	1 (1.1)	NA		1 (1.38)	NA	
Health Insurance	Insured	61 (100)	50 (90.3)	11 (9.74)	NA	41 (69.09)	20 (30.91)	NA
	Uninsured	0 (0)	(0.00)	(0.00)		0 (0.00)	(0.00)	
Zip code	Urban	56 (81.8)	45 (80.14)	11 (96.95)	**0.023**	38 (83.54)	18 (77.83)	0.768
	Rural	7 (18.2)	6 (19.86)	1 (3.05)		4 (16.46)	3 (22.17)	
Clinical factors								
Family history of cancer	Yes	57 (94.0)	47 (94.11)	10 (92.68)	0.824	38 (98.38)	19 (84.02)	**<0.001**
	No	6 (6.0)	4 (5.89)	2 (7.32)		4 (1.62)	2 (15.98)	
Personal history of cancer	Yes	23 (79.7)	19 (92.78)	4 (29.46)	**<0.0001**	16 (82.50)	7 (71.58)	0.512
	No	12 (20.3)	5 (7.22)	7 (70.54)		8 (17.50)	4 (28.42)	
Psychological factors								
Cancer worry	Not at all or Slightly	7 (8.8)	4 (7.43)	3 (20.24)	0.1936	3 (7.35)	4 (11.89)	0.198
	Somewhat	19 (33.0)	18 (36.33)	1 (5.75)		13 (25.25)	6 (49.38)	
	Extremely or Moderately	35 (58.2)	27 (56.25)	8 (74.01)		25 (67.40)	10 (38.73)	
Perceived genetic susceptibility	A little or Not at all	60 (95.4)	48 (94.85)	12 (100.00)	NA	39 (93.29)	21 (100.00)	NA
	A lot or Somewhat	3 (4.6)	3 (5.15)	NA		3 (6.71)	NA	
Importance of genetics for cancer prevention	A little or Not at all	58 (92.5)	47 (93.64)	11 (81.82)	**0.019**	40 (92.97)	18 (91.36)	0.719
	A lot or Somewhat	4 (7.5)	3 (6.36)	1 (18.18)		2 (7.03)	2 (8.64)	
Importance of genetics for early detection of cancer	A little or Not at all	61 (97.6)	49 (97.29)	12 (100.00)	NA	42 (100.00)	19 (92.06)	NA
	A lot or Somewhat	2 (2.4)	2 (2.71)	NA		NA	2 (7.94)	
Fatalistic belief	A little or Not at all	43 (72.8)	36 (73.27)	7 (68.99)	0.83	28 (66.09)	15 (86.94)	0.131
	A lot or Somewhat	18 (27.2)	13 (26.73)	5 (31.01)		13 (33.91)	5 (13.06)	
Prevention not possible	Strongly/Somewhat disagree	10 (21.1)	8 (22.48)	2 (10.01)	0.339	6 (12.20)	4 (39.94)	0.083
	Strongly/Somewhat agree	51 (78.9)	41 (77.52)	10 (89.99)		35 (87.80)	16 (60.06)	
Too many recommendations	Strongly/Somewhat disagree	41 (73.0)	33 (73.41)	8 (70.00)	0.862	29 (71.59)	12 (76.09)	0.767
	Strongly/Somewhat agree	20 (27.0)	16 (26.59)	4 (30.00)		12 (28.41)	8 (23.91)	
Self-efficacy	Strongly/Somewhat disagree	52 (83.2)	41 (82.90)	11 (85.33)	0.882	35 (85.32)	17 (78.63)	0.671
	Strongly/Somewhat agree	9 (16.8)	8 (17.10)	1 (14.67)		6 (14.68)	3 (21.37)	
Desire to know about mutation status	A little or Not at all	57 (88.7)	45 (87.49)	12 (100.00)	NA	41 (94.22)	16 (75.67)	0.204
	A lot or Somewhat	5 (11.3)	5 (12.51)	NA		1 (5.78)	4 (24.33)	

HCP: healthcare provider, GC: genetic counselor; FDR: first-degree relative; wtd: weighted; NA: not applicable; significant results are in bold.

**Table 2:** Logistic regression of sharing high-risk cancer genetic test results.

Variable	Shared Result with HCP/GC (n = 51, 90.2%)			Shared Result with FDR (n = 42, 69.3%)		
	**OR**	**95% CI**	***p***	**OR**	**95% CI**	***p***
Race/ethnicity						
Non-Hispanic White	ref	-	-	ref	-	-
Other	4.987	0.24–102.56	0.291	0.476	0.03–6.64	0.574
Sex						
Male	ref	-	-	ref	-	-
Female	**9.322**	**1.93–45.08**	**0.006**	1.252	0.03–59.51	0.907
Family history of cancer						
No/Not sure	ref	-	-	ref	-	-
Yes	3.71	0.07–186.33	0.504	6.194	0.31–293.37	0.347
Personal history of cancer						
No cancer history	Ref	-	-	ref	-	-
Yes	**0.025**	**0.01–0.09**	**<0.0001**	1.917	0.34–10.79	0.453
Income						
Less than $50,000	ref	-	-	ref	-	-
More than $50,000	0.981	0.14–7.01	0.984	1.297	0.04–38.35	0.878

HCP: healthcare provider, GC: genetic counselor; FDR: first-degree relative; significant results are in bold.

**Table 3:** Descriptive characteristics of individuals who shared results from genetic health risk testing.

Variable	Categories	Sharing with HCP/GC			Sharing with FDR		
		Shared	Did Not Share	*p*-Value	Shared	Did Not Share	*p*-Value
Age. y	Mean, SD	50.1, 2.2	59.1, 2.3	0.005 *	49.8, 2.9	53.7, 3.7	0.43 *
		**n (wtd%)**	**n (wtd%)**		**n (wtd%)**	**n (wtd%)**	
Sex	Male	4 (9.0)	3 (2.7)	0.29 ^	3 (4.95)	4 (6.73)	0.19 ^
	Female	47 (81.2)	9 (7.1)		39 (64.32)	17 (24.00)	
Income	Less than $20,000	5 (10.1)	2 (0.72)	NA ^	5 (10.10)	2 (0.72)	0.80 ^
	$20,000 to less than $35,000	4 (3.4)	1 (0.42)		4 (3.35)	1 (0.42)	
	$35,000 to less than $50,000	4 (9.7)	0 (0)		4 (9.73)	0 (0)	
	$50,000 to less than $75,000	9 (28.9)	4 (4.01)		9 (28.89)	4 (4.01)	
	$75,000 or more	28 (38.0)	3 (2.23)		28 (38.03)	3 (2.23)	
Education	Less than high school	2 (6.2)	1 (0.30)	NA ^	2 (4.30)	1 (2.17)	0.99 ^
	12 years/Completed high school	9 (19.6)	0 (0)		6 (13.73)	3 (5.83)	
	College graduate or higher	19 (46.5)	3 (3.97)		14 (34.94)	8 (15.50)	
	Some college	20 (17.9)	7 (4.89)		19 (15.67)	8 (7.11)	
Race/Ethnicity	Non-Hispanic White	39 (82.8)	9 (8.92)	NA ^	34 (67.47)	14 (24.21)	NA ^
	Non-Hispanic Black	2 (2.1)	1 (0.44)		1 (0.44)	2 (2.11)	
	Hispanic/Latino	5 (4.8)	0 (0)		3 (3.12)	2 (1.66)	
	Non-Hispanic Asian	1 (1.0)	0 (0)		1 (0.99)	0 (0)	
	Non-Hispanic other	0 (0)	0 (0)		0 (0)	0 (0)	
Health Insurance	Insured	50 (90.3)	11 (9.74)	NA ^	41 (69.09)	20 (30.91)	NA ^
	Uninsured	0 (0)	0 (0)		0 (0)	0 (0)	
Zip code	Urban	45 (72.3)	11 (9.49)	0.13 ^	38 (57.87)	18 (23.92)	0.78 ^
	Rural	6 (17.9)	1 (0.30)		4 (11.40)	3 (6.81)	

HCP: healthcare provider, GC: genetic counselor; FDR: first-degree relative; wtd: weighted; * *t*-test; ^ chi-squared test.

**Table 4:** Descriptive characteristics of individuals who shared results from ancestry genetic testing.

Variable	Categories	Sharing with HCP/GC			Sharing with FDR		
		Shared	Did Not Share	*p*-Value	Shared	Did Not Share	*p*-Value
Age, y	Mean, SD	49.0, 5.1	52.1, 1.4	0.56 *	50.8, 1.6	57.0, 2.4	0.05 *
		**n (wtd%)**	**n (wtd%)**		**n (wtd%)**	**n (wtd%)**	
Sex	Male	4 (1.38)	127 (45.42)	0.51 ^	96 (36.46)	35 (10.34)	0.43 ^
	Female	9 (2.79)	157 (50.41)		129 (43.87)	37 (9.33)	
Income	Less than $20,000	2 (0.28)	14 (5.98)	NA ^	12 (4.37)	4 (1.89)	0.90 ^
	$20,000 to less than $35,000	3 (0.34)	16 (6.12)		15 (4.74)	4 (1.72)	
	$35,000 to less than $50,000	1 (0.20)	40 (12.37)		31 (10.05)	10 (2.52)	
	$50,000 to less than $75,000	3 (1.29)	51 (15.75)		39 (13.23)	15 (3.80)	
	$75,000 or more	4 (2.07)	142 (52.90)		114 (45.89)	32 (9.08)	
Education	Less than high school	0 (0)	1 (0.16)	NA ^	0 (0)	1 (0.16)	NA ^
	12 years/Completed high school	1 (0.66)	32 (16.28)		24 (13.44)	9 (3.51)	
	College graduate or higher	7 (1.65)	171 (40.63)		136 (35.25)	42 (7.03)	
	Some college	5 (1.86)	77 (38.37)		62 (31.25)	20 (8.98)	
Race/Ethnicity	Non-Hispanic White	8 (2.86)	208 (80.94)	NA ^	169 (68.60)	47 (15.20)	0.19 ^
	Non-Hispanic Black	2 (0.40)	26 (5.43)		21 (4.82)	7 (1.01)	
	Hispanic/Latino	2 (0.91)	25 (5.28)		20 (5.56)	7 (0.63)	
	Non-Hispanic Asian	1 (0.06)	6 (1.81)		3 (0.66)	4 (1.22)	
	Non-Hispanic other	0 (0)	9 (2.31)		4 (0.48)	5 (1.83)	
Health Insurance	Insured	13 (4.17)	278 (94.04)	NA ^	221 (78.68)	70 (19.54)	0.53 ^
	Uninsured	0 (0)	4 (1.79)		2 (1.62)	2 (0.17)	
Zip code	Urban	12 (2.98)	257 (83.05)	0.56 ^	205 (69.38)	64 (16.64)	0.82 ^
	Rural	1 (1.19)	27 (12.78)		20 (10.94)	8 (3.03)	

HCP: healthcare provider, GC: genetic counselor; FDR: first-degree relative; wtd: weighted; * *t*-test; ^ chi-squared test.

## Data Availability

The data are publicly available through the National Health Information National Trends Survey website.
